# Iatrogenic Hepatocellular Adenoma in Relation to an Unusual Complication of Cardiac Surgery

**DOI:** 10.1016/j.jaccas.2025.106668

**Published:** 2026-01-28

**Authors:** Luis E. Echeverria, Omar Nicolas Ferro-Peñuela, Angie Yarlady Serrano-García, Laura V. Arciniegas-Landinez

**Affiliations:** a Heart Failure and Cardiac Transplant Division, Fundación Cardiovascular de Colombia, Universidad de Santander, Santander, Colombia; b Department of Medicine, Universidad de Santander, Santander, Colombia; c Department of Clinical Trials, Fundación Cardiovascular de Colombia, Santander, Colombia; d Medical School, Universidad Industrial de Santander, Santander, Colombia

**Keywords:** adenoma, Budd-Chiari syndrome, echocardiography, liver cell, liver transplantation

## Abstract

**Background:**

Inferior vena cava (IVC) obstruction is a rare but serious complication after atrial septal defect (ASD) repair.

**Case Summary:**

A 50-year-old woman with prior ASD closure presented for liver transplant evaluation due to cirrhosis and a suspected hepatocellular adenoma. Examination revealed congestive hepatopathy and dyspnea (NYHA II). Imaging identified IVC obstruction from Eustachian valve entrapment and thrombus at the cavoatrial junction. She underwent successful IVC recanalization and thrombectomy, with resolution of symptoms, prompting suspension of transplant listing.

**Discussion:**

IVC obstruction after ASD repair is exceedingly rare but can mimic liver disease and precipitate Budd-Chiari syndrome. Unlike previously reported cases, our patient uniquely demonstrated the coexistence of cirrhosis and an HNF1A-inactivated hepatocellular adenoma, a combination not described before in this setting. This case underscores the need to consider cardiac causes in unexplained hepatopathy, especially post-ASD repair.

**Take-Home Messages:**

IVC obstruction should be suspected in post-ASD repair patients with hepatopathy. Early recognition allows curative intervention and avoids unnecessary transplantation.

## Visual Summary

**Figure:**
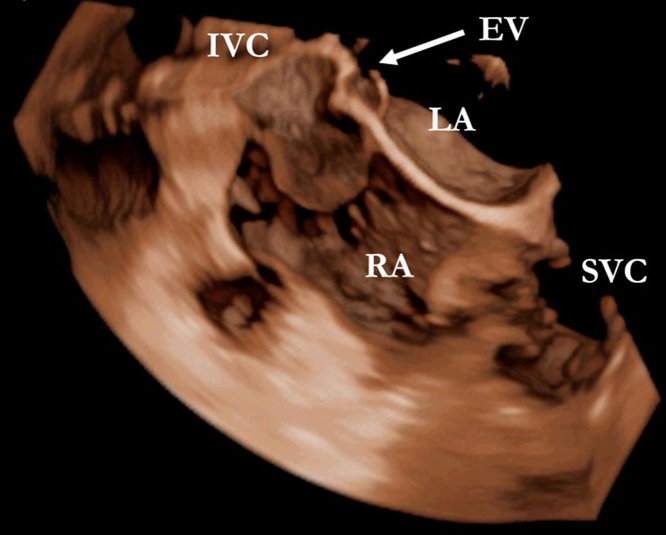

**Visual Summary:**
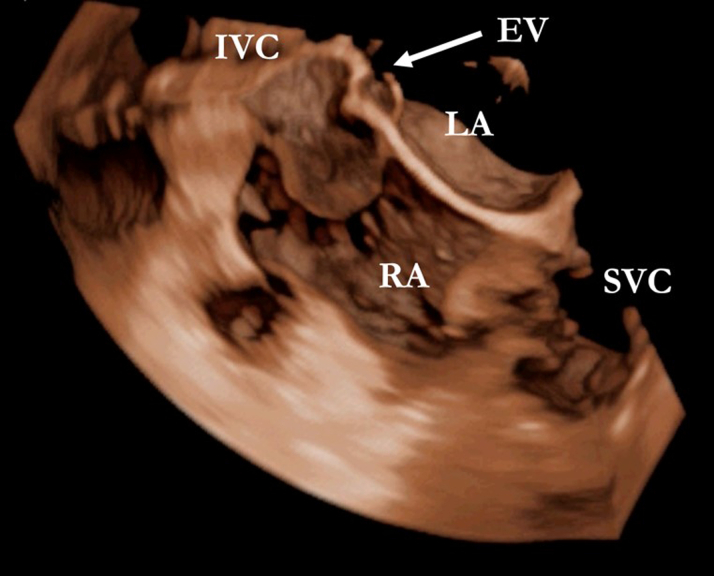
Transesophageal Echocardiogram With a 3-Dimensional Image Showing the Eustachian Valve Entrapped in the Atrial Septum

## History of Presentation

**Figure 1:**
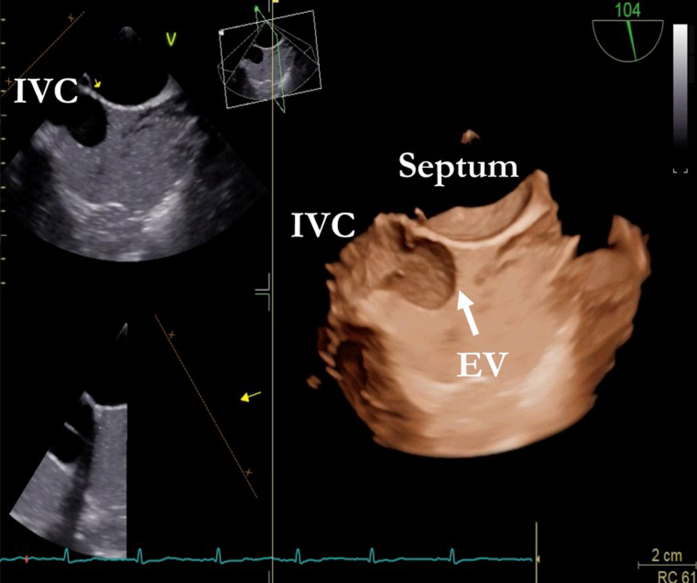
Venous Saline Contrast Shows the Separation of the Right Atrium by the Eustachian Valve (EV) and the Isolation of the Inferior Vena Cava (IVC)

**Figure 2:**
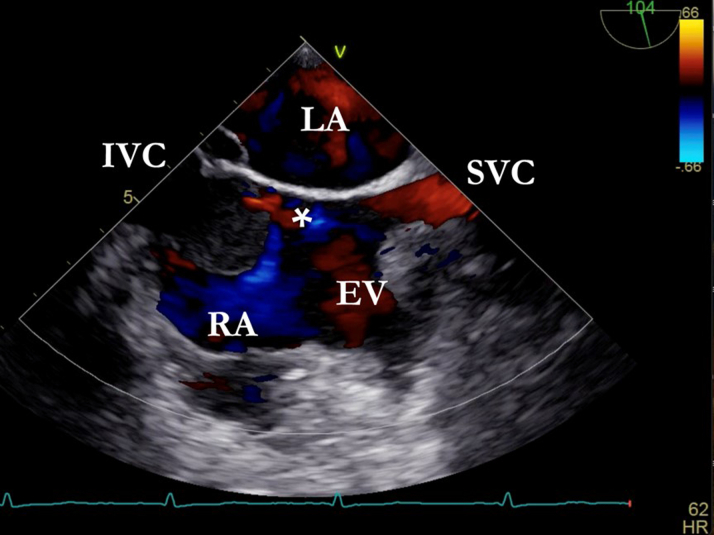
Bicaval 2-Dimensional Image Where, in Addition to the Previously Mentioned Findings, a Small Communication Through the Membrane Is Observed∗ EV = Eustachian valve; IVC = inferior vena cava; LA = left auricle; RA = right auricle; SVC = superior vena cava.

A 50-year-old woman with a history of atrial septal defect (ASD) repair via thoracotomy at age 43 was admitted for pre–liver transplant evaluation due to cirrhosis and a suspected hepatocellular carcinoma (HCC). Laboratory tests revealed normal liver function and tumor markers, except for mild thrombocytopenia. Physical examination showed stigmata of congestive hepatopathy and dyspnea on moderate exertion, classified as NYHA functional class II. Transesophageal echocardiography (TEE) performed to assess residual ASD incidentally revealed a membrane in the right atrium, originating from the Eustachian crest and separating the inferior vena cava (IVC), with no contrast passage (Figures 1 and 2).

**Take-Message Messages**

• Inferior vena cava obstruction is an uncommon but clinically significant complication following atrial septal defect closure.
• Assessment of inferior vena cava obstruction should be considered in patients presenting with congestive symptoms, vascular abnormalities, or a history of cardiac surgery.
• Postoperative assessment using transesophageal echocardiography may be considered in patients who have undergone atrial septal defect closure.

## Past Medical History

**Figure 3:**
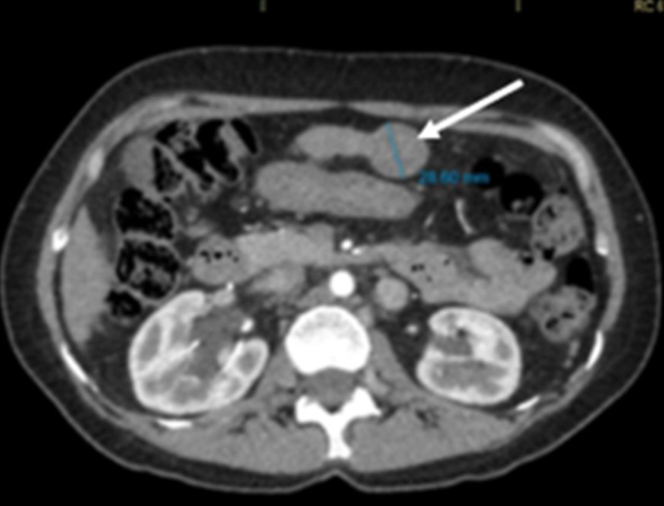
Hepatocellular Adenoma Originating From Hepatic Segment III

She underwent ASD closure at age 43 for lifelong exertional dyspnea. Two years prior to presentation, she developed lower-limb edema and progressive dyspnea. A computed axial tomography (CT) scan of the abdomen and pelvis (total abdomen) showed a heterogeneous solid expansive lesion observed in segment III of the liver, measuring 2.8 cm in diameter. It demonstrated heterogeneous enhancement after contrast injection, with rapid washout appearing hypodense on venous and delayed phase images (Figure 3). Hepatospecific magnetic resonance imaging revealed cirrhosis and a 3-cm LI-RADS 4 nodule in segment III, suggestive of HCC, prompting transplant evaluation.

## Differential Diagnosis

The differential diagnosis for this case included HCC, portal vein thrombosis, and right ventricular dysfunction.

## Investigations

Cavography through a dilated azygos vein demonstrated complete IVC obstruction at its junction with the right atrium, with normal right-sided pressures. Chest CT angiography identified a hypodense, pseudodiverticular structure from the IVC into the right atrium, suggesting a redundant Eustachian valve (EV).

## Management

The patient underwent successful IVC recanalization, thrombectomy, and cavoatrial junction repair. Intraoperatively, an entrapped EV was identified at the prior atriotomy site, along with organized thrombi within the IVC. The procedure included right atriotomy with removal of the thrombi, resection of the entrapped EV, and repair of the cavoatrial junction. Concomitant mitral valve annuloplasty with ring implantation was also performed. Systemic anticoagulation with unfractionated heparin was administered intraoperatively, and the patient was transitioned to oral anticoagulation with warfarin for 3 months.

Following symptomatic improvement and clarification of the underlying cause, liver transplantation was deferred. Subsequently, a segmentectomy revealed a cirrhotic-appearing liver with a micronodular surface and a 3-cm segment III lesion. Histopathological analysis confirmed a hepatocellular adenoma (HCA; HNF1A-inactivated subtype).

## Discussion

ASD is a common congenital anomaly, typically asymptomatic and often diagnosed incidentally.^1^ Surgical correction is associated with low mortality but carries complication rates of approximately 1% (major) and 5% (minor), including IVC obstruction, a rare event that can lead to Budd-Chiari syndrome (BCS). BCS results from partial or complete obstruction of hepatic venous outflow, impairing blood drainage and causing portal hypertension, cirrhosis, and, in some cases, precancerous or malignant liver lesions.^2^ Genetic mechanisms, including HNF1A inactivation, STAT3 activation, and β-catenin pathway mutations (CTNN1 exon 3), have been implicated in HCA formation.^3^

The EV is an embryologic remnant located at the IVC orifice, directing fetal blood toward the foramen ovale. Although it often persists into adulthood, it has no defined function in this period. Entrapment or dysfunction of the EV can impede venous return and raise central venous pressure, contributing to BCS.^4^

Similar cases have been reported in the literature. The first, described by Frank et al in 1980,^5^ involved the detection of a murmur along the upper sternal border, which ultimately led to the identification of an IVC thrombus caused by the EV closing against the atrial septum. Years later, Diegeler et al^6^ described a patient presenting with congestive heart failure 20 years after ASD closure, in whom a contracted Teflon patch and calcified tissue nearly occluded the IVC entry into the right atrium. Patel et al^7^ reported a woman who, 2 days after ASD repair, exhibited high-velocity residual flow in the posterolateral right atrium on echocardiography; CT confirmed a 5-cm IVC thrombus attributed to inadvertent EV suturing. Similarly, Akpinar et al^4^ described a case where a new postoperative murmur prompted TEE, revealing an obstructive thrombus related to EV entrapment.

Unlike previously reported cases,^4^^,^^8^ our patient did not present with acute symptoms following ASD repair. The diagnosis of IVC obstruction was established by TEE and confirmed by CT angiography during the workup of a suspected HCC. In this case, IVC flow obstruction manifested as BCS caused by EV entrapment at the atriotomy site and associated thrombi, ultimately resulting in cirrhosis without hepatic dysfunction, a hallmark of BCS-related cirrhosis.^2^ The presumptive diagnosis of HCC was based on magnetic resonance imaging, abdominal CT, and clinical history, in line with current guidelines.^9^ However, differentiating HCC from benign regenerative lesions, such as HCA, remains challenging because both demonstrate arterial-phase hyperenhancement on contrast-enhanced imaging. In such cases, follow-up imaging, alternative contrast agents, or histopathological confirmation may be required, the latter of which established the final diagnosis of HCA in our patient.^9^

HCA-like nodules have been described in the setting of BCS. Ibarrola et al reported 6 cases of BCS complicated by secondary HCA, potentially related to altered portal venous perfusion and compensatory hyperarterialization.^10^ Notably, none of those cases occurred in the context of cirrhosis, which is frequently observed in BCS and was present in our patient. To date, no published reports have directly linked IVC obstruction to HCA development.

Our case is, to our knowledge, the first to describe the coexistence of cirrhosis and a genetically characterized HNF1A-inactivated HCA in the setting of chronic IVC obstruction following ASD repair, which highlights its novelty and clinical relevance. We hypothesize that chronic IVC obstruction from EV entrapment increased hepatic venous pressure, disrupted venous outflow, and promoted the formation of HCA, consistent with prior associations between hepatic vascular disorders and hepatocellular nodules.^3^ However, we acknowledge that this represents an association based on pathophysiological plausibility rather than definitive causality and should be interpreted with caution.

## Limitations

This report describes a single patient, which precludes establishing a definitive causal relationship between IVC obstruction and HCA, and the findings may not be generalizable. However, the coexistence of cirrhosis and an HNF1A-inactivated adenoma in this context has not been previously reported and provides clinically relevant insights that may guide evaluation of similar cases.

## Follow-Up

After discharge, the patient experienced recovery of her functional class and edema. She continued with periodic follow-ups by cardiology and hepatology for 12 months. During this period, she remained in NYHA functional class I, with stable liver function tests (including bilirubin, aminotransferases, alkaline phosphatase, and INR) and no evidence of recurrent obstruction or new hepatic lesions on ultrasound and CT imaging. Long-term hepatology follow-up confirmed the absence of recurrence of HCA or hepatic decompensation.

## Conclusions

EV entrapment in the atrial septum during ASD repair is an exceedingly rare complication, with only a few cases reported in the literature. It can lead to BCS with significant hemodynamic and hepatic repercussions. Early diagnosis is critical due to the condition's potential reversibility and should be considered in patients with hepatopathy and a history of ASD repair.

## Funding Support and Author Disclosures

The authors have reported that they have no relationships relevant to the contents of this paper to disclose.
